# Clinical and radiographic outcomes after pulpotomies using mineral trioxide aggregate mixed with distilled water or 2.25% sodium hypochlorite gel: a randomized controlled clinical trial

**DOI:** 10.1038/s41405-025-00356-2

**Published:** 2025-07-25

**Authors:** Mawia Karkoutly, Amjad Abu Hasna, Ok Hyung Nam, Ricardo Machado, Saleh Al Kurdi, Nada Bshara

**Affiliations:** 1 Department of Pediatric Dentistry, Faculty of Dentistry, Damascus University, Damascus, Syrian Arab Republic; 2 Department of Restorative Dentistry, Endodontics Division, Institute of Science and Technology, São Paulo State University (ICT-UNESP) Eng. Francisco José Longo Avenue 777, São José dos Campos, São Paulo Brazil; 3 School of Dentistry, Universidad Espíritu Santo, Samborondón, Ecuador; 4 Department of Pediatric Dentistry, School of Dentistry, Kyung Hee University, Seoul, Korea; 5 Department of Pediatric Dentistry, Kyung Hee University College of Dentistry, Kyung Hee University Medical Center, Seoul, Korea; 6 Department of Restorative Sciences, Division of Endodontics, College of Dentistry, Health Sciences Center, University of Oklahoma, Oklahoma City, OK USA; 7 Department of Orthodontics and Pediatric Dentistry, Faculty of Dentistry, Arab International University, Daraa, Syrian Arab Republic

**Keywords:** Paediatric dentistry, Pulp conservation

## Abstract

**Objectives:**

Sodium hypochlorite (NaOCl) gel is an effective additive for white mineral trioxide aggregate (WMTA) that enhances its chemical and physical characteristics and significantly reduces setting time while maintaining biocompatibility. The study sought to compare the outcomes of pulpotomies performed in primary teeth using WMTA mixed with 2.25% NaOCl gel or WMTA mixed with distilled water (DW).

**Materials and methods:**

It was a randomized, triple-blinded, split-mouth, controlled clinical trial. Forty primary second molars from cooperative children aged 5–10 who required pulpotomy were randomly assigned to the control group—WMTA  + DW or experimental group—WMTA + NaOCl gel for clinical and radiographic assessments at 3, 6, and 12 months of follow-up. The clinical evaluation involved checking for spontaneous pain, pain on percussion, tooth mobility, abscesses, sinus tracts, and the exfoliation of the treated tooth. The periapical radiographic evaluation identified whether there was furcal radiolucency, internal or external root resorption, and widening of the periodontal ligament. Canal obliteration was not considered a failure.

**Results:**

The WMTA + NaOCl gel group showed a 90% and 95% clinical and radiographical success rate, respectively, at 3, 6, and 12 months of follow-up. In the control group, the clinical success rate was 90% after 3, 6, and 12 months, whereas the radiographical success rate was 100% at 3 months and decreased to 95% after 6 and to 85% after 12 months. No statistically significant difference was detected between the two groups (*p* > 0.05).

**Conclusions:**

The present research validated WMTA + NaOCl gel as a potentially effective material for pulpotomy in primary molars.

## Introduction

Dental caries remains a significant public health challenge due to its complex etiology, high incidence, widespread prevalence, and management difficulties [1–3]. Despite inadequate eating and hygiene habits being the main contributing factors to the occurrence of dental caries, the composition of the oral microbiota also plays a key role [4, 5]. Since untreated lesions usually progress, leading to irreversible destruction of enamel, and dentin tissues, and to pulp tissues inflammation. Dental caries is the main factor responsible for premature teeth loss [6] resulting in eating, nutritional, phonetic, esthetic, and psychological problems in children [1–3]. Consequently, different approaches to maintaining young participants’ teeth have been studied [7, 8].

Pulpotomy is a reliable and effective treatment for managing pulp-exposed primary teeth caused by carious lesions or traumatic injuries. This approach is particularly efficient as the coronal pulp tissue often harbors microorganisms and exhibits signs of inflammation and degeneration. During the procedure, the affected coronal pulp is excised, and a biocompatible material is applied to the remaining healthy pulp tissue to promote healing and preserve tooth function [7, 9].

For many years, calcium hydroxide [Ca(OH)_2_] was the most widely used material to perform pulpotomies [10, 11]. However, Ca(OH)_2_ solubility, lack of adhesion, and insufficient mechanical properties encouraged the search and development of different alternatives like calcium silicate-based cements including mineral trioxide aggregate (MTA) [12, 13], Biodentine [14], and Five Mineral Oxide cement (5MO) [15, 16].

MTA is a powder mainly composed of calcium disilicate, it was initially developed for sealing root perforations after mixing it with distilled water (DW) [12, 17–19]. Among favorable properties of MTA, biocompatibility, sealing ability, low solubility and long-term stability stand out [17–19]. However, MTA has some drawbacks, such as high cost, hard handling due to its inadequate consistency, long setting time, and reduced working time when mixed with DW [17–19].

To address these drawbacks, researchers have explored different vehicles and additives to improve MTA’s working properties. Sodium hypochlorite (NaOCl) gel has been identified as an effective additive for MTA that enhances its chemical and physical characteristics, improves handling, increases antibacterial properties, and significantly reduces setting time while maintaining biocompatibility. According to Jafarnia et al. [20] and Alnezi et al. [21], MTA combined with 3% NaOCl gel is biocompatible and may be a suitable substitute for DW. Moreover, Karkoutly et al. [7] found that mixing white mineral trioxide aggregate (WMTA) with 2.25% NaOCl gel improved odontoblastic integrity in human primary molars during short- and medium-term assessments. Furthermore, at 28 days, WMTA exhibited similar chemical composition, surface morphology, and alkalinity compared to MTA mixed with DW.

Based on scientific dynamics, randomized controlled clinical trials are invariably necessary to prove the effectiveness of a material or technique [22]. Thus, this study sought to compare the outcomes of pulpotomies performed in primary teeth using WMTA mixed with 2.25% NaOCl gel or WMTA mixed with DW. The null hypothesis was that the WMTA mixed with 2.25% NaOCl gel did not improve the clinical and radiographic outcomes compared to WMTA mixed with DW.

## Materials and methods

### Study design and sample size calculation

The study was a randomized, triple-blinded, single-center, split-mouth, active-controlled clinical trial of two groups. It was conducted at the Department of Pediatric Dentistry, Faculty of Dentistry, Damascus University, from January 2024 to January 2025. The sample size was determined using G*Power version 3.1.9.4 (G*Power 3.1.9, Heinrich Hein Universität Düsseldorf, Düsseldorf, Germany). With an 80% power (1-β error probability) and a significance level (α error probability) of 5%, the final sample included 40 teeth, with 20 teeth assigned to each study group.

### Ethical considerations

The Biomedical Research Ethics Committee approved the trial (1254/2024), and it was registered retrospectively at the ISRCTN registry (ISRCTN27453183) on 23/01/2025. The trial was conducted by the Consolidated Standards of Reporting Trials (CONSORT) guidelines [23] and the World Medical Association Declaration of Helsinki, as updated in 2013 [24]. The medical procedure was thoroughly explained, and written informed consent was obtained by acquiring the signature of the participant’s legally authorized representatives before enrollment. Legal guardians have the right to withdraw consent at any time. Each child received complete dental care. There was no discrimination in participant enrollment based on ethnicity, gender, or socioeconomic status. Participants’ data was kept confidential.

### Participant enrollment and grouping

#### Inclusion criteria

- Cooperative children aged between 5 and 10 years.
- Deep caries extend into pulp proximity or cause mechanical exposure during excavation.
- Provoked pain only resolves immediately after stimulus removal.
- No pathologic root resorption.
- Radiographic confirmation that the roots are not excessively resorbed, with at least 1/2 to 2/3 of root length remaining.
- Children requiring pulpotomy treatment on their second primary molars [25].

#### Exclusion criteria

- Uncooperative children.
- Children with systematic diseases and/or allergies to the anesthetic agents.
- Children younger than 5, or older than 10 years.
- Teeth with >1/3 of root length lost or irregular/internal resorption.
- Children with compromised conditions or exhibiting signs and symptoms indicative of pulp necrosis, as well as a history of spontaneous or nocturnal pain in their second primary molars [25].

Two experienced pediatric dentists (intraclass correlation coefficient >0.8) selected 20 participants from 31 who were referred to the Department of Pediatric Dentistry. The selected participants, who had 40 s primary molars that required pulpotomy, were randomly assigned to one of two groups (*n* = 20) according to the pulp dressing material used:

- Group 1 (WMTA + NaOCl gel): WMTA (Rootdent, TehnoDent Co., Belgorod, Russia) mixed with 2.25% NaOCl gel (LET’S CLEAN Concentrated Chlorine, DTIC®, Damascus, Syria) was categorized as the interventional group.
- Group 2 (WMTA + DW): WMTA combined with DW was designated the control group [7].

### Randomization and blinding

The study was a triple-blinded trial, ensuring that the dentist, participants, and outcome assessors were unaware of the group assignments. A blinded investigator implemented a simple randomization method, using a coin toss for each participant. Subsequently, the second primary molars were allocated at random to either the control group or the intervention group for each participant, using a split-mouth design. For each participant, a coin toss determined whether the left or right second primary molar, in either the upper or lower arch, would receive the intervention, WMTA + NaOCl gel, or the control, WMTA + DW. The contralateral molar, opposite side, same arch, automatically received the alternative treatment, ensuring a direct within-patient comparison.

### Treatment procedure

A diagnostic periapical radiograph was obtained using an intraoral periapical sensor (i-sensor, Guilin Woodpecker Medical Instrument Co., LTD., Guilin, China). Following the administration of sufficient anesthesia and achieving proper isolation, the decay was excavated, and a coronal pulpotomy was carried out. Hemorrhage was managed by applying a sterile cotton pellet soaked in normal saline and exerting pressure on the pulp stump for 5 min. In the control group, WMTA powder was combined with distilled water at a ratio of 3:1, powder-to-liquid, and subsequently, a 3 mm thick layer of MTA was applied to the pulp. In the interventional group, WMTA was mixed with a 2.25% NaOCl gel at a 3:1 ratio of powder to gel. A stainless-steel crown (SSC) (Kids Crown, Shinhung, Seoul, Korea) was the final restoration and immediately delivered after the treatment [7]. Follow-up Intervals were planned at 3, 6, and 12 months [26]. Radiographs were taken at each interval, and radiographic changes became apparent three months post-pulpal treatment.

### Outcome measures

The clinical evaluation involved checking for spontaneous pain, pain on percussion, tooth mobility, abscesses, sinus tracts, and the exfoliation of the treated tooth. The periapical radiographic evaluation identified whether there was furcal radiolucency, internal or external root resorption, and widening of the periodontal ligament [27]. Canal obliteration was not considered a failure [28]. Outcome measures were assessed by two investigators who were blinded to the treatment (intraclass correlation coefficient > 0.8). The outcome assessors were calibrated by averaging the scores provided by the assessors evaluating the children.

### Statistical analysis

Data was analyzed using IBM SPSS software version 24 (IBM SPSS Statistics® version 24, IBM Corp., New York, USA). Descriptive statistics were presented as frequency and percentage. The chi-square test was used to analyze categorical data. A significance level of *p* < 0.05 was established for statistical significance.

## Results

**Fig. 1:**
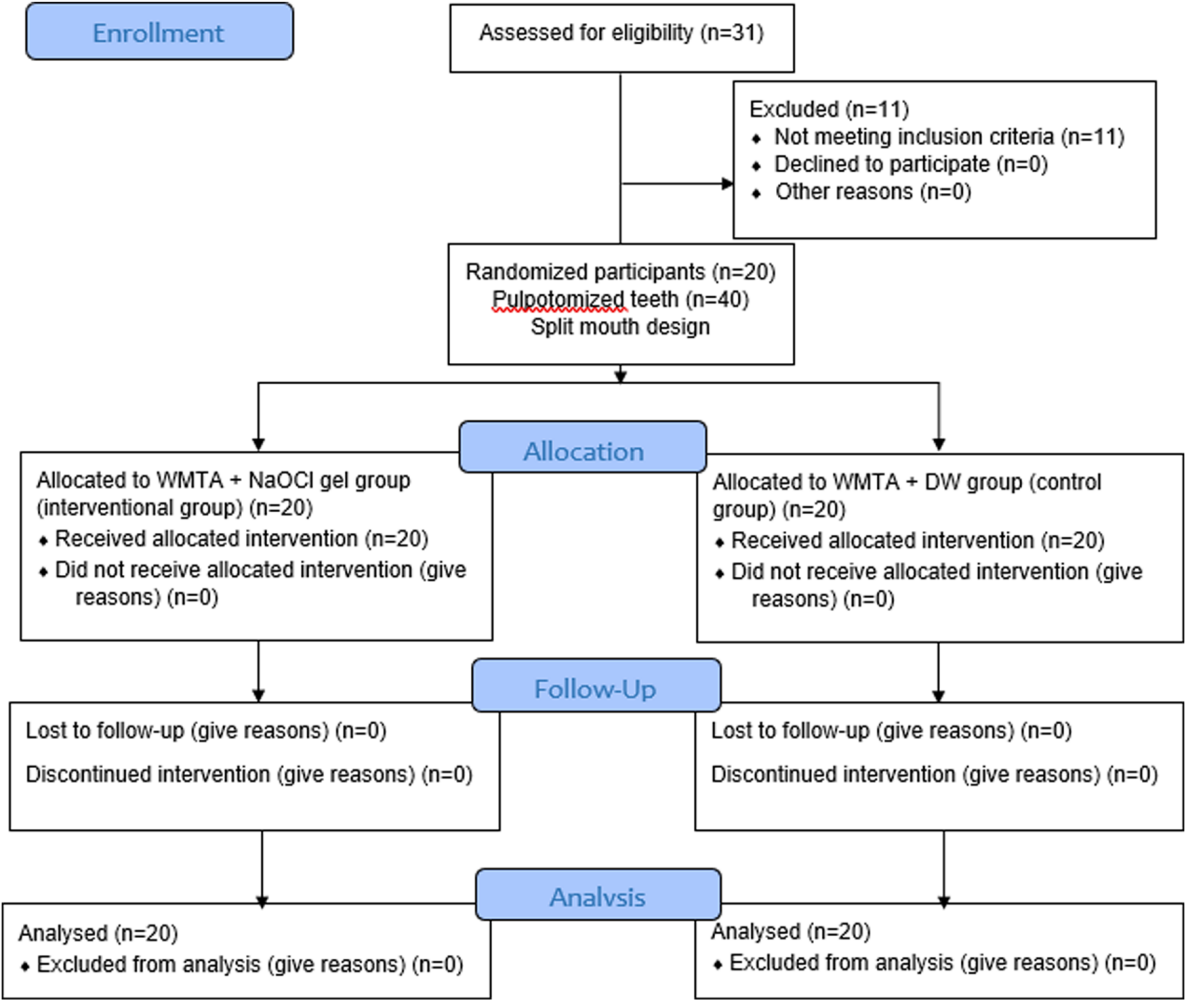
CONSORT flowchart.

Figure 1 displays the CONSORT flowchart. Over half (65.00%) of the participants were males, with an average age of 7.42 years (SD 1.23; range 5–10 years), as shown in Table 1.

**Table 1:** Baseline demographic characteristics for the participants.

Characteristics	*N* = 20
Sex	
Female n (%)	7 (35.00)
Male n (%)	13 (65.00)
Age (years)	
Mean ± SD	7.42 ± 1.23
Min—Max	5–10

Both MTA + NaOCl gel and MTA + DW groups demonstrated a 90% success rate in clinical outcomes after three months of follow-up, with two cases (10.0%) reporting pain on percussion in both groups (Table 2). Furthermore, MTA + NaOCl gel group’s radiographic success rate was 95%, as one case (5.0%) showed internal root resorption, external root resorption, and widening of the periodontal ligament (Table 2).

**Table 2:** Comparing clinical and radiographic criteria at 3-months follow-up.

Follow-up interval	Variables		MTA + NaOCl gel N/Total (%)	MTA + DW N/Total (%)	*p*value
3 months	Clinical criteria	Spontaneous pain	0/20 (0.0%)	0/20 (0.0%)	–
		Pain on percussion	2/20 (10.0%)	2/20 (10.0%)	1.000
		Tooth mobility	0/20 (0.0%)	0/20 (0.0%)	–
		Abscess	0/20 (0.0%)	0/20 (0.0%)	–
		Sinus tract	0/20 (0.0%)	0/20 (0.0%)	–
		Exfoliation	0/20 (0.0%)	0/20 (0.0%)	–
	Success rate		18/20 (90%)	18/20 (90%)	1.000
	Radiographic criteria	Furcal radiolucency	0/20 (0.0%)	0/20 (0.0%)	–
		Internal root resorption	**1/20 (5.0%)**	0/20 (0.0%)	0.311
		External root resorption	**1/20 (5.0%)**	0/20 (0.0%)	0.311
		widening of the periodontal ligament	**1/20 (5.0%)**	0/20 (0.0%)	0.311
	Success rate		19/20 (95%)	20/20 (100%)	0.311

Values in bold refer to a case with multiple outcome failures, estimated as 1/20 (5.0%).

Conversely, MTA + DW group achieved a 100% radiographical success rate at the three-month follow-up (Table 2). At a 6-month follow-up, the clinical success rate remained 90% for both groups. However, in MTA + DW group, one case (5.0%) experienced internal and external root resorption, resulting in a radiographical success rate of 95%.

**Fig. 2:**
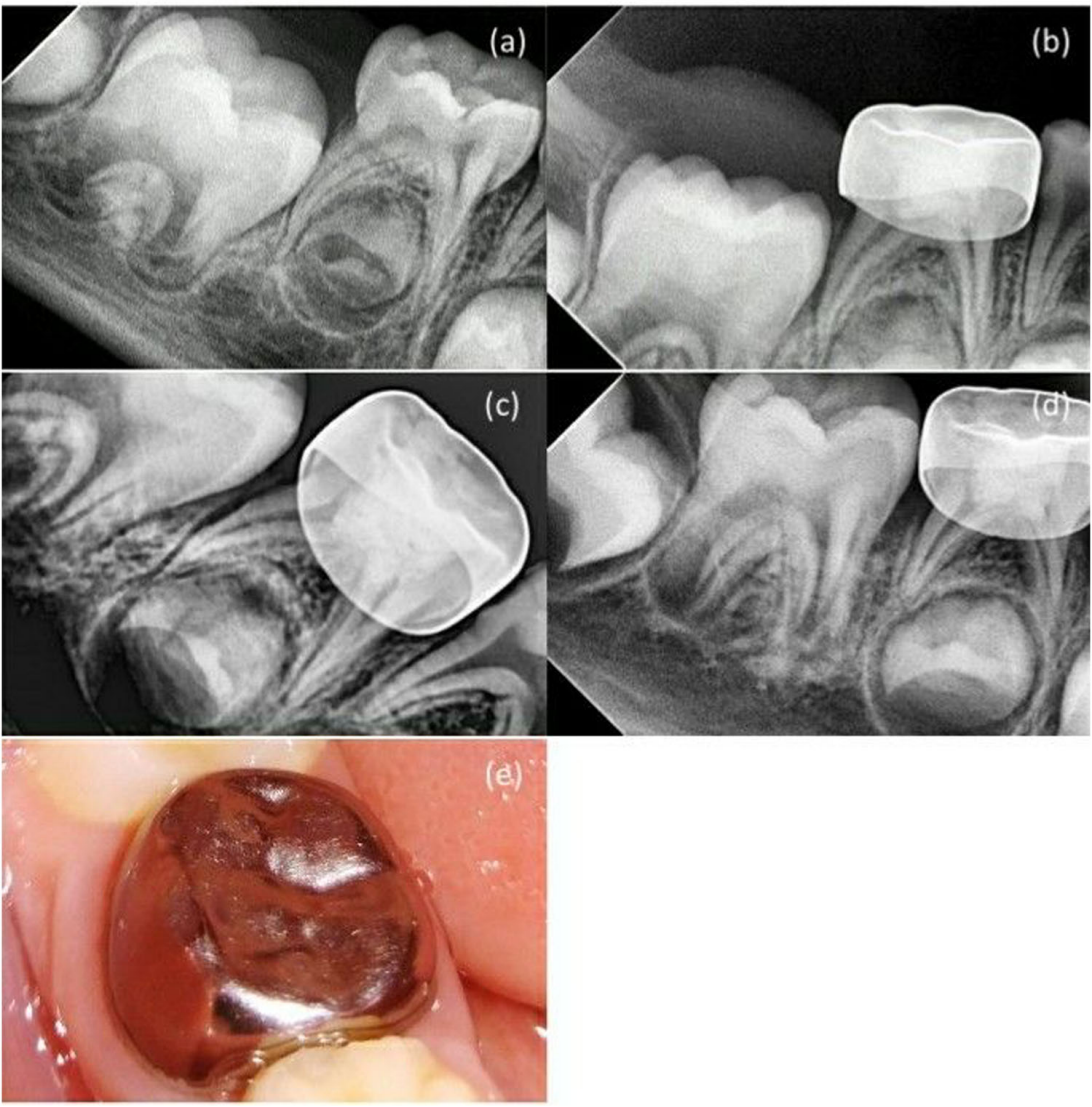
MTA + NaOCl gel group in second primary molar pulpotomy in a female participant aged 5 years. **a** A preoperative radiograph. **b** 3-month follow-up. **c** 6-month follow-up. **d** 12-month follow-up. **e** A photograph after 12-month follow-up.

**Fig. 3:**
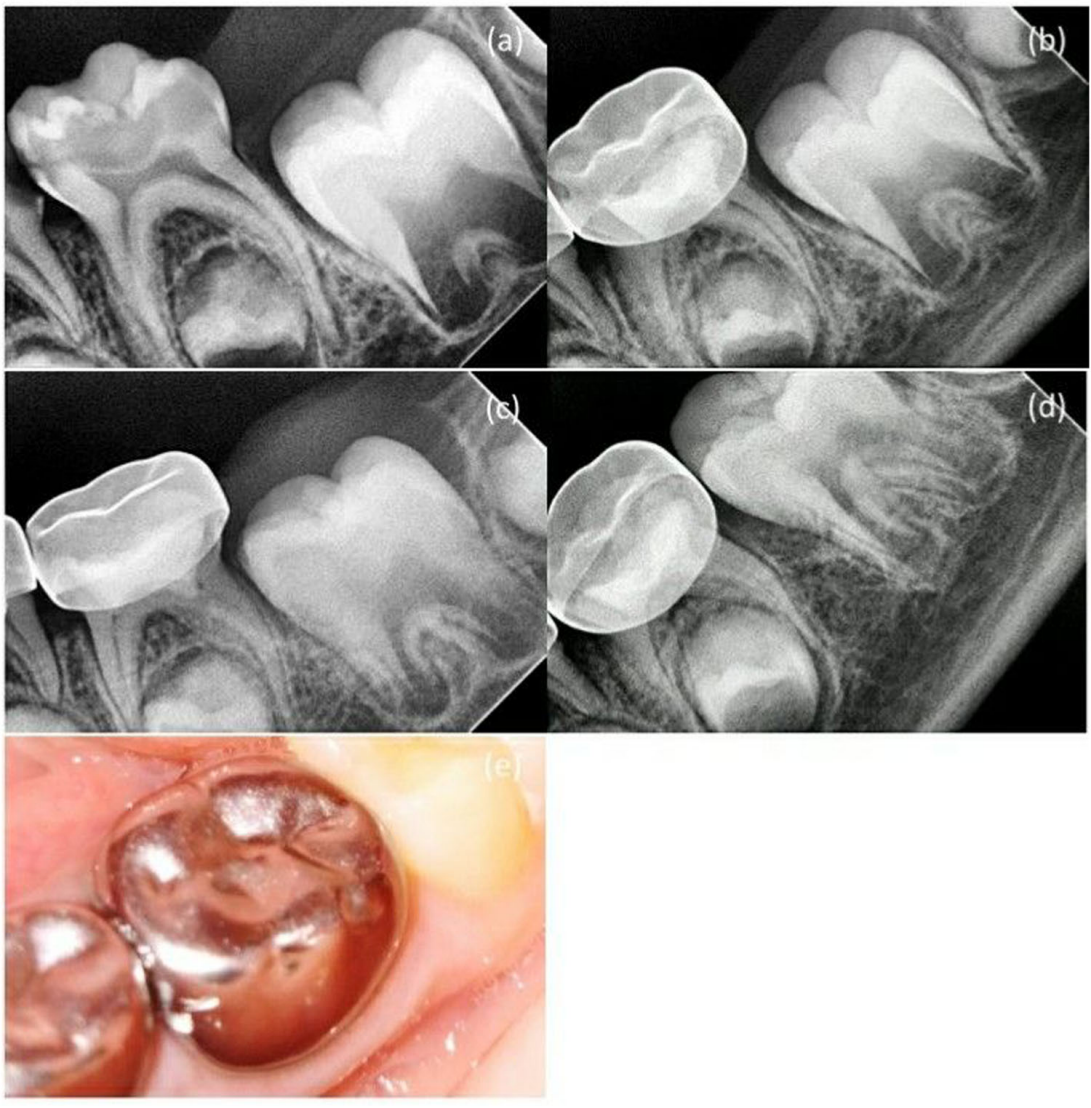
MTA + NaOCl gel group in second primary molar pulpotomy in a female participant aged 5 years. **a** A preoperative radiograph. **b** 3-month follow-up. **c** 6-month follow-up. **d** 12-month follow-up. **e** A photograph after 12-month follow-up.

**Fig. 4:**
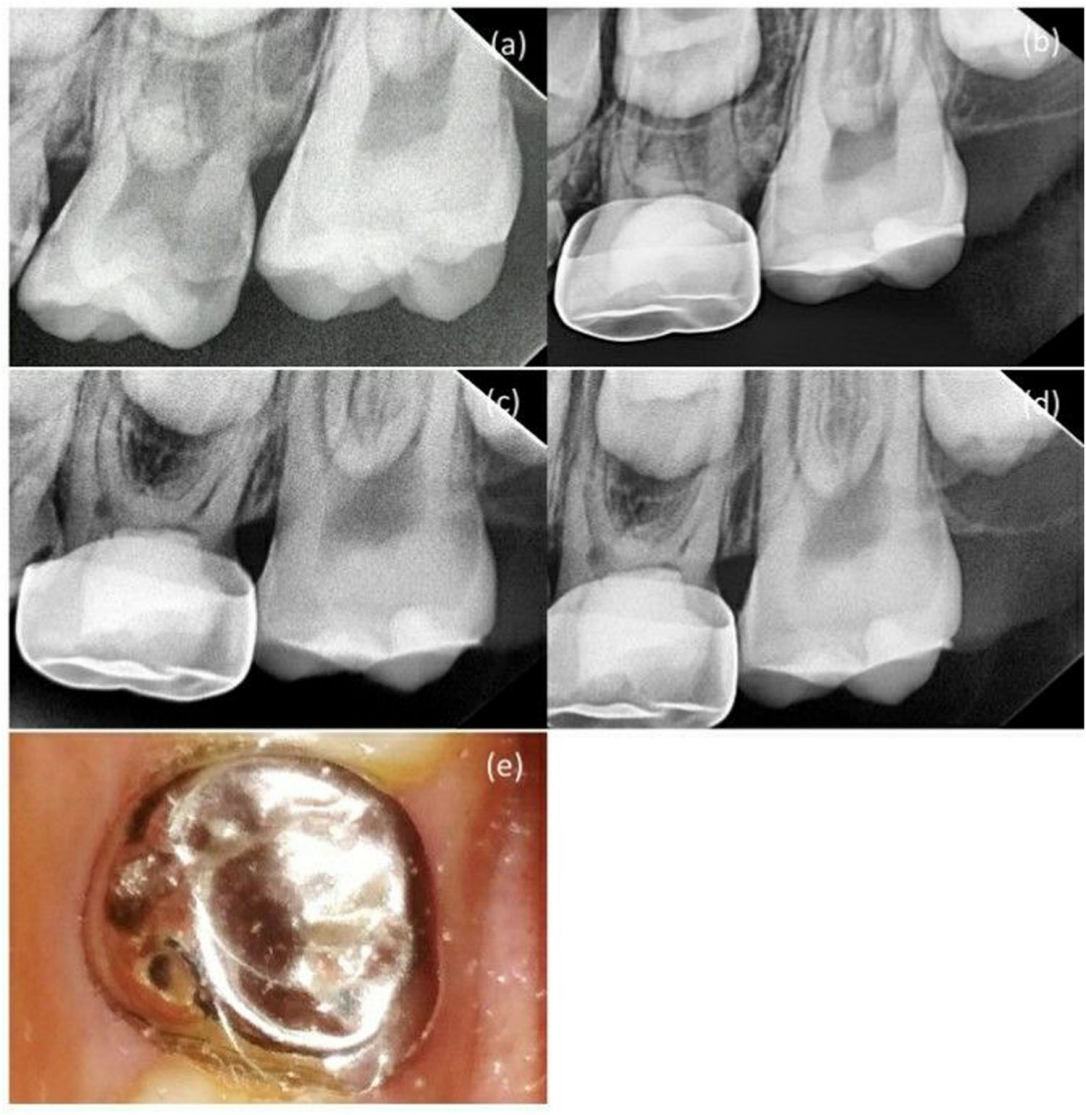
MTA + DW group in second primary molar pulpotomy in a male participant aged 8 years. **a** A preoperative radiograph. **b** 3-month follow-up. **c** 6-month follow-up. **d** 12-month follow-up. **e** A photograph after 12-month follow-up.

Furthermore, in MTA + NaOCl gel group, a canal obliteration was detected in one case which was not regarded as a radiographic failure. At the 12-month follow-up, the clinical success rate for both groups remained to be 90%, while the radiographic success rate for MTA + NaOCl gel group also remained at 95%. In contrast, MTA + DW group experienced a decrease in radiographic success rate to 85% due to the diagnosis of internal root resorption in two cases (10.0%). Nevertheless, no statistically significant difference was observed in the clinical and radiographic success rates between the two groups at different follow-up intervals (*p* > 0.05) (Tables 2,  3,  4), suggesting that the MTA + DW group did not outperform the MTA + NaOCl gel group in improving treatment outcomes (Figs. 2, 3, 4).

**Table 3:** Comparing clinical and radiographic criteria at 6-months follow-up.

Follow-up interval	Variables		MTA + NaOCl gel N/Total (%)	MTA + DW N/Total (%)	*p*value
6 months	Clinical criteria	Spontaneous pain	0/20 (0.0%)	0/20 (0.0%)	–
		Pain on percussion	2/20 (10.0%)	2/20 (10.0%)	1.000
		Tooth mobility	0/20 (0.0%)	0/20 (0.0%)	–
		Abscess	0/20 (0.0%)	0/20 (0.0%)	–
		Sinus tract	0/20 (0.0%)	0/20 (0.0%)	–
		Exfoliation	0/20 (0.0%)	0/20 (0.0%)	–
	Success rate		18/20 (90%)	18/20 (90%)	1.000
	Radiographic criteria	Furcal radiolucency	0/20 (0.0%)	0/20 (0.0%)	–
		Internal root resorption	**1/20 (5.0%)**	**1/20 (5.0%)**	1.000
		External root resorption	**1/20 (5.0%)**	**1/20 (5.0%)**	1.000
		widening of the periodontal ligament	**1/20 (5.0%)**	0/20 (0.0%)	0.311
	Success rate		19/20 (95%)	19/20 (95%)	1.000

Values in bold refer to a case with multiple outcome failures, estimated as 1/20 (5.0%).

**Table 4:** Comparing clinical and radiographic criteria at 12-months follow-up.

Follow-up interval	Variables		MTA + NaOCl gel N/Total (%)	MTA + DW N/Total (%)	*p*value
12 months	Clinical criteria	Spontaneous pain	1/20 (5.0%)	1/20 (5.0%)	1.000
		Pain on percussion	2/20 (10.0%)	2/20 (10.0%)	1.000
		Tooth mobility	1/20 (5.0%)	1/20 (5.0%)	1.000
		Abscess	0/20 (0.0%)	1/20 (5.0%)	0.311
		Sinus tract	0/20 (0.0%)	0/20 (0.0%)	–
		Exfoliation	0/20 (0.0%)	0/20 (0.0%)	–
	Success rate		18/20 (90%)	18/20 (90%)	1.000
	Radiographic criteria	Furcal radiolucency	0/20 (0.0%)	0/20 (0.0%)	1.000
		Internal root resorption	**1/20 (10.0%)**	2/20 (10.0%) **1/20 (5.0%)**	0.292
		External root resorption	**1/20 (5.0%)**	**1/20 (5.0%)**	1.000
		widening of the periodontal ligament	**1/20 (10.0%)**	**1/20 (5.0%)**	1.000
	Success rate		19/20 (95%)	17/20 (85%)	0.605

Values in bold refer to a case with multiple outcome failures, estimated as 1/20 (5.0%).

## Discussion

While MTA is recognized as the optimal dressing material for pulpotomy in primary molars, which serves as a reference for comparison, its prolonged setting time is a disadvantage when mixing WMTA with DW [17–19]. Consequently, it has been suggested to combine MTA with different accelerators, such as NaOCl gel. Jafarani et al. [20] investigated the cytotoxicity of a mixture of WMTA and 3% NaOCl gel by assessing its impact on the survival of mouse fibroblast cells (L929) over 24, 48, and 72 h and found that the mixture is biocompatible. Karkoutly et al. [7] performed a histological investigation on human primary molars, indicating that the combination of WMTA and 2.25% NaOCl gel exhibited superior odontoblastic integrity short-term and mid-term without any signs of pulp necrosis. Furthermore, while the initial physical and chemical properties of the NaOCl gel group were worse, they became comparable after one month based on surface morphological and chemical evaluations [7]. To the author’s knowledge, no study in the reviewed literature has evaluated the clinical and radiographic effectiveness of the mixture in human primary molars, emphasizing the need for such studies. As a result, the objective of this study is to perform a clinical and radiographic evaluation of pulpotomy in primary teeth using WMTA mixed with 2.25% NaOCl gel.

The present research utilized a split-mouth design to minimize variability, as each participant serves as their control [29]. Children aged 5 to 10 years were selected for this study because they cooperate during dental procedures [30]. Moreover, pulpotomies of primary molars are commonly performed in this age group [31]. In the present study, pulpotomy performed with MTA was set as a control group since many studies have deemed it the gold standard for comparison with other materials [32–34]. According to Karygianni’s study [35], the gel form was preferred over the solution form because mixing a 2.25% NaOCl solution with MTA produced toxic effects and compromised biocompatibility. In this study, a 2.25% NaOCl gel was selected over lower concentrations due to its efficacy in tissue dissolution and its alkaline properties [36]. Andrade et al. [37] indicated that mixing WMTA with a 1% NaOCl gel did not enhance its antibacterial effectiveness when compared to using a mixture of WMTA and distilled water (DW). In addition, a 2.5% NaOCl gel has shown efficacy in reducing the levels of *Enterococcus faecalis* [38]. Moreover, the study by Al Kurdi et al. [39] revealed that mixing 2.2% NaOCl gel with white Portland cement improved its antibacterial characteristics, as white Portland cement shares a similar chemical composition with WMTA, consisting of 75% white Portland cement. In this research, the inclusion criteria were followed rigorously, ensuring that the pulp condition was suitable for pulpotomy. The literature identifies the two primary causes of pulpotomy failures in primary teeth as incorrect diagnosis of radicular pulp inflammation before treatment and contamination of the pulp resulting from microleakage [37, 38]. Thus, primary molars showing indications and symptoms of pulp necrosis, along with a history of spontaneous or nocturnal pain, were not included [40, 41]. In addition, Stainless-steel Crowns (SSCs) were chosen for the final restoration because of their effective biological seal, which is pivotal for successful treatment outcomes [40, 42]. SSCs are often favored for restorations in the posterior teeth since their characteristics align closely with the functional performance of natural dentition. Moreover, their key benefits are improved durability, increased longevity, and a reduced rate of recurrent caries [40, 43].

The results of the current study suggested that at 12-month follow-up, one case exhibited furcal radiolucency and internal and external root resorption in both study groups. However, the MTA + DW group reported three cases with external root resorption and one with abscess. Pathological resorption can also occur in primary teeth when the pulp is infected. The bone destruction associated with pathological resorption causes rapid root loss of the primary tooth, leading to premature tooth loss [44]. This finding contrasts with many studies [45–49], which suggested no external or internal root resorption was detected in the MTA + DW group. However, as reported by Godgi et al. [34], one tooth exhibited internal resorption at a 3-month follow-up, which did not advance to cause further perforation of the root over the subsequent 9 months. Comparable observations were noted in two cases during a period of 25–38 months in a study conducted by Holan et al. [46] and after 12 months in the Jabbarifar et al. [50] study. The result could explained by the fact that the WMTA + NaOCl gel mixture exhibited higher antibacterial efficacy. According to Al Kurdi et al. [39], mixing white Portland cement with 2.2% NaOCl gel enhanced its antibacterial efficacy against *E. Faecalis*. In addition, according to Karkoutly et al. [7], the WMTA mixed with 2.25% NaOCl gel started with a pH of 9.7 during the initial 24 h and rose to 11.6 after 28 days. Furthermore, the WMTA mixed with DW recorded a pH of 8.5, which also increased to 11.1, demonstrating that both combinations resulted in an alkaline pH. However, the MTA combined with 2.25% NaOCl gel showed greater alkalinity, contributing to its antibacterial efficacy and thereby improving treatment results [7]. Teeth that didn’t respond well to treatment and showed outcome failures were either treated with root canal therapy or extracted.

The study found that the WMTA + NaOCl gel group showed canal obliteration in one case, indicating tooth vitality rather than failure, as it resulted from odontoblast activity [51, 52]. Histological findings supported this, with initial odontoblastic disorganization at 7 days but normal tissue structure after 90 days [7]. The group also exhibited small calcifications, 87.5% at 7 days, reducing to single calcifications by 90 days, unlike the control group, which showed no pulp calcification [7]. The WMTA + NaOCl gel performed comparably to WMTA + DW, with no significant difference in treatment outcomes [7]. Despite initial inferior properties, the NaOCl gel achieved similar performance after one month [7]. Thus, the null hypothesis was not rejected, confirming that both materials behaved similarly. Zero drop-outs do not mean zero failures. No patients were lost to follow-up. Teeth extracted for resorption were counted as failures, not drop-outs, especially with strict inclusion criteria.

A limitation of the current study is the follow-up assessment duration of only 12 months. Typically, longer evaluation periods are preferable to assess the success rate of pulpotomy materials [40]. Therefore, it is recommended that trials with extended follow-up durations and larger sample sizes be performed to ascertain findings.

## Conclusions

Based on our findings, both the WMTA + DW group and the WMTA + NaOCl gel group achieved favorable treatment outcomes. Therefore, the present research validated WMTA + NaOCl gel as a potentially effective material for pulpotomy in primary molars. The potential benefits of substituting DW with NaOCl gel in clinical practice include no risk of necrosis, improved handling characteristics, and a shorter setting time, an essential requirement in pediatric dentistry.

## Data Availability

The datasets generated during and/or analyzed during the current study are available from the corresponding author on reasonable request.
